# Polyphenols in Metabolic Diseases

**DOI:** 10.3390/molecules27196280

**Published:** 2022-09-23

**Authors:** Amin Gasmi, Pavan Kumar Mujawdiya, Sadaf Noor, Roman Lysiuk, Roman Darmohray, Salva Piscopo, Larysa Lenchyk, Halyna Antonyak, Kateryna Dehtiarova, Mariia Shanaida, Alexandr Polishchuk, Volodymyr Shanaida, Massimiliano Peana, Geir Bjørklund

**Affiliations:** 1 Société Francophone de Nutrithérapie et de Nutrigénétique Appliquée, 69100 Villeurbanne, France; 2 Laboratoire Interuniversitaire de Biologie de la Motricité, Université Claude Bernard, 69100 Villeurbanne, France; 3 Birla Institute of Technology and Science-Pilani, Hyderabad 500078, India; 4 Institute of Molecular Biology and Biotechnology, Bahauddin Zakariya University, Multan 60800, Pakistan; 5 Department of Pharmacognosy and Botany, Danylo Halytsky Lviv National Medical University, 79010 Lviv, Ukraine; 6 CONEM Ukraine Life Science Research Group, Danylo Halytsky Lviv National Medical University, 79010 Lviv, Ukraine; 7 Institute for Advanced Training of Pharmacy Specialist, National University of Pharmacy, 61001 Kharkiv, Ukraine; 8 CONEM Ukraine Pharmacognosy and Natural Product Chemistry Research Group, National University of Pharmacy, 61002 Kharkiv, Ukraine; 9 Department of Ecology, Ivan Franko National University of Lviv, 79005 Lviv, Ukraine; 10 Department of Pharmacognosy and Medical Botany, I. Horbachevsky Ternopil National Medical University, 46001 Ternopil, Ukraine; 11 Design of Machine Tools, Instruments and Machines Department, Ternopil Ivan Puluj National Technical University, 46001 Ternopil, Ukraine; 12 Department of Chemical, Physics, Mathematics and Natural Sciences, University of Sassari, Via Vienna 2, 07100 Sassari, Italy; 13 Council for Nutritional and Environmental Medicine (CONEM), 8610 Mo i Rana, Norway

**Keywords:** phenolic compounds, natural sources, metabolic syndrome, bioprotective property, therapeutic effect

## Abstract

Polyphenols (PPs) are a large group of phytochemicals containing phenolic rings with two or more hydroxyl groups. They possess powerful antioxidant properties, multiple therapeutic effects, and possible health benefits in vivo and in vitro, as well as reported clinical studies. Considering their free-radical scavenging and anti-inflammatory properties, these substances can be used to treat different kinds of conditions associated with metabolic disorders. Many symptoms of metabolic syndrome (MtS), including obesity, dyslipidemia, atherosclerosis, elevated blood sugar, accelerating aging, liver intoxication, hypertension, as well as cancer and neurodegenerative disorders, are substantially relieved by dietary PPs. The present study explores the bioprotective properties and associated underlying mechanisms of PPs. A detailed understanding of these natural compounds will open up new opportunities for producing unique natural PP-rich dietary and medicinal plans, ultimately affirming their health benefits.

## 1. Introduction

Metabolic diseases such as hyperglycemia, obesity, dyslipidemia, and hypertension are now considered global problems of the world population [1,2]. Their occurrence increases yearly, and nowadays, they are considered a significant danger for human beings as the most prevalent disorders worldwide [3].

Metabolic syndrome (MtS) is a complex coexisting diagnosis including abdominal obesity, increased blood pressure, elevated fasting glucose, reduced high-density lipoprotein-cholesterol levels, and elevated triglyceride levels [4]. MtS increases the risk factors of cardiovascular diseases (CVD), which puts huge pressure on the healthcare economy of the whole society [4]. Therefore, it is an urgent challenge for researchers to determine active pharmaceutical ingredients to improve MtS and its complications.

Mts have a complex etiology, including several pathophysiological mechanisms and factors that may cause the development of MtS, such as genetics, lifestyle, diet, and gut microbiome state [5]. The molecular changes result from the interaction between environmental and genetic factors, and oxidative stress and systemic inflammation significantly contribute to MtS pathogenesis [1]. Thus, an effective method of combating MtS comprises not only appropriate diet and physical activity but also consuming drugs and/or food supplementation. Therefore, discovering new natural substances that could reduce the symptoms of MtS through antioxidant and anti-inflammatory effects and the ability to normalize lipid and carbohydrate metabolism are very important issues for researchers and clinicians [1].

Oxidative stress and inflammation are common pathophysiology keys involved in the development and progression of metabolic disorders [6]. Thus, finding appropriate natural compounds to mitigate metabolic disorder’s symptoms and prevent related diseases’ progression is necessary.

In recent years, polyphenols (PPs) have been considered the key plant-based bioactive compounds against various diseases, including metabolic diseases, cardiovascular and neurodegenerative disorders, and some varieties of cancer [7,8]. PPs are getting much attention in the medical industry to treat different metabolic diseases due to their intrinsic antioxidant and anti-inflammatory properties [9,10].

**Figure 1:**
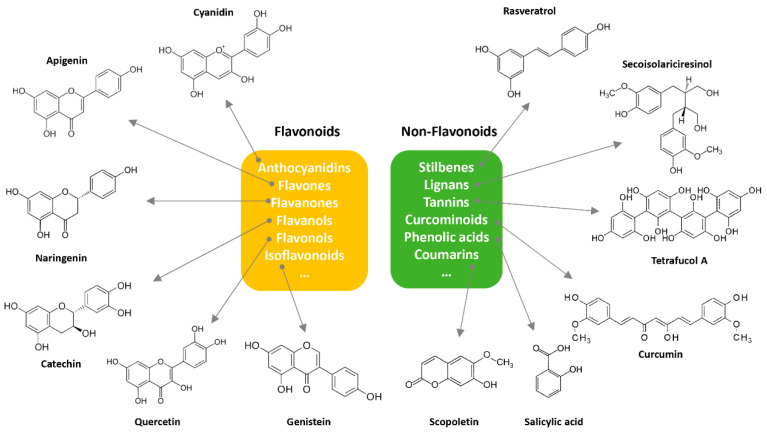
Classification of PPs with some representative examples.

About eight thousand polyphenolic compounds have been recognized nowadays [11,12]. PPs are naturally occurring complexes in various plants, including herbs, tea, fruits, and vegetables. In plants, these compounds play a key role in pigmentation, growth, ultraviolet rays protection, and against pathogens. PPs are also an essential part of various chemical industries for producing commodity chemicals, food additives, cosmetics, and paints [13]. Generally, PPs are known as plant secondary metabolites and can be categorized by the presence of several phenolic groups [14]. These compounds are classified based on the chemical complexity of their respective phenolic structure (flavonoids and non-flavonoids, Figure 1) [15].

It is interesting to note that polyphenolic compounds share a similar chemical structure. The structure of naturally occurring PPs varies from simple molecules (phenolic acid) to complex molecules (tannins) depending on the length of the chain attached [16]. PPs occur primarily in conjugated form, with one or more sugar residues attached to hydroxyl groups, although direct linkages to an aromatic carbon atom can be found [17,18]. PPs can also be found in associations with other compounds, such as amines, carboxylic and organic acids, and lipids; also common are their linkages with other phenols [18].

As PPs are found abundantly in the plant kingdom, their consumption in the human diet is not much surprising. These compounds are found in almost every balanced diet, especially where fruits and vegetables are mostly consumed [19]. However, quantitative information on PPs for various foods is unavailable because of their diverse nature and other factors responsible for altering their concentration in the diet. PPs possess similar properties, but their complex linkage with other compounds makes their separation challenging. Ample research has been done on different samples from various sources (foods, beverages, and plants). Thus, a deep understanding of these naturally occurring compounds and their associated biosynthesis will open new avenues to design special dietary plans enriched with PPs that ultimately strengthen respective health benefits. Considering the antioxidant and anti-inflammatory features of PPs, these compounds can be employed to treat various diseases associated with metabolic disorders [1].

Treatments of MtS may involve the design of natural drugs and special food products enriched with high-quality PPs or by the controlled drug and/or supplement delivery system, which can lead to the health improvement of the human population. Besides this, Siroma et al. supposed that scientific studies regarding ingestion of PPs and other natural nutrients could improve global food education in different countries to help schools, families, and businesses to reduce obesity, hyperglycemia, and other metabolic diseases [20].

This review aims to provide deep insight into the effect of different PPs on preventing and healing major metabolic disorders through reviewing the results of different preclinical and clinical studies. It should be noted that intact delivery of PPs to specific organs and tissues is challenging, which causes their poor bioavailability and ultimately reduces their potential benefits. Therefore, this review also describes experimental studies to highlight the importance of nanotechnology-based delivery systems to enhance target delivery and bioavailability. Furthermore, this review also highlights the importance of the chemical structure of PPs in their biological activities. To the best of our knowledge, this is the first review article that summarized the beneficial effects of polyphenols in metabolic disorders along with better ways of their effective delivery and interconnectedness of chemical property information.

## 2. Polyphenols in the Prevention and Treatment of Different Metabolic Disorders

### 2.1. Oxidative Stress and Inflammation

Oxidative stress is one of the health conditions which occurs due to an imbalance of free radicals (oxygen or nitrogen species) and the defensive ability of the body to respond to reactive species to heal the respective disorder [21]. The production of significant reactive oxygen (ROS) and nitrogen species is one of the consequences of the normal functioning of alive intracellular structures [22]. The structural damage of various proteins, cell tissues, permeable membranes, and nucleic acids is due to exposure to highly reactive oxygen-based species such as hydrogen peroxide and superoxide anions [23]. To counter this oxidative stress, body cells continuously express several species, such as enzymes, to detoxify the resulting reactive species and ultimately heal the damage in the respective region. Species released by the body cells to encounter oxidative stress may come from enzymes, bacterial cells, mammalian cells, and PPs. In oxidative stress, the mechanism of cell damage occurs due to the different chemical actions of oxygen-based free radicals [24]. These reactive species may come from various sources by the abnormal metabolic system (aerobic metabolism) that may generate undesirable reactive species and cause cell death. It is found that the intake of antibiotics is also a source of generating these species [25,26].

#### 2.1.1. Pre-Clinical Studies

The damaging effects of free radicals are counteracted by the organism’s antioxidant defenses leading to redox homeostasis [27]. Oxidative stress occurs if the antioxidant defense cannot counteract a high production of free radicals. As a result of the depletion of intracellular antioxidants, consuming exogenous sources of molecules with antioxidant properties such as PPs, coenzyme Q, vitamin C, or vitamin E is needed.

The antioxidant effects of PPs are mainly due to their redox potential, which allows them to act as hydrogen donors or chelating agents of metal ions [27]. The use of PPs as a redox-active species is well established [28]. Researchers have reported that PPs possess antioxidative properties and can easily suppress oxidative stress, possibly resulting in further inflammation. Generally, PPs scavenge most free radicals directly or suppress free radical production by inhibiting NADPH oxidases and xanthine oxidase [29]. Many studies showed that phytochemical substances enriched with PPs are excellent antioxidants to fight against free radicals such as ROS. Thus, it is suggested that food rich (1 g/day) in phenolic-based compounds is more favorable for disease prevention in human life [30]. Many researchers have reported using cocoa-based PPs and their associated flavonoids to modulate oxidative stress [31]. However, the information on daily intake for these PPs is not exactly available. Thus careful mechanistic studies are required to quantify these PPs in the human diet [6]. Lignin is another source of rich PPs from sesame seed; oils have been reported for antioxidative properties. However, the reason behind their actual antioxidant properties is still unclear [32].

Other foods, such as black rice, which contains the C-glycosyl flavonoid, and black tea, with its phenolic pigments, are excellent for antioxidative properties [33]. From these studies, we can conclude that PPs from various dietary sources can suppress the evolution of numerous diseases in the body and provide a healthy metabolic system due to their featured antioxidative properties.

The antioxidant power of phenolic compounds is substantially proven [34]. Generally, the beneficial role of PPs is attributed to their antioxidant effect able to inactivate reactive oxidant species [35]. Overproduction of ROS can be associated with the nuclear factor-κB-mediated inflammatory process in obesity [36]. Thus, such famous dietary sources of PPs as green tea, chocolate, coffee, red wine, and various fruits and vegetables are highly recommended as natural antioxidants.

Inflammation is a health condition in which blood cells provide a defensive response to protect the body from foreign substances [37]. In simple terms, it is an automatic response of our body’s defense system to an external substance with irritating capacities. The causes of inflammation may include the attack of pathogens such as viruses, injuries from external sources, such as scrapes, and irritation from chemicals or radiation on the skin [38,39].

Inflammation can be categorized from simple to severe and may even cause serious health conditions to the normal human body. In this regard, our body also prepares particular responses according to the reaction required by the respective region. In severe inflammation, the body generally reacts in the form of a fever, indicating that the body is preparing an active response to the foreign pathogen. The body’s response may also include changes in blood composition. It is also found that inflammation is not always favorable for the body. Sometimes, it leads to severe health conditions (bowel disease, rheumatoid arthritis) during the fight against pathogens [40].

As is known, inflammation is key in developing chronic diseases, including diabetes, cancer, arthritis, and cardiovascular and neurodegenerative diseases [41]. In this regard, the biological activities of PPs are found to be very beneficial for treating various kinds of inflammation either by dietary plans or by using external drug delivery enriched with PPs [42]. Zamani-Garmsiri et al. described the significant role of PPs, such as caffeic, chlorogenic, and ellagic acids, genistein, and silymarin, in decreasing low-grade chronic systemic inflammation and related pathways [43]. Berry-derived PPs managed the inflammation via different mechanisms, particularly inhibiting the transcriptional factor nuclear factor-κB (NF-κB) [44]. Since honey contains a distinctive amount of phenolic compounds, there is an intense interest in their effects on inflammation-mediated diseases [41,45].

Various studies have reported the contribution of PPs in immunity response (modulation in cytokines and gene expression) against inflammation [46]. For example, resveratrol originated from grapes can modulate cytokines and suppress inflammatory disease. In another study, researchers reported that curcumin from turmeric and mustard plants is a source of a non-flavonoid polyphenol that can effectively suppress inflammatory mediators such as cyclooxygenase (COX) [47,48,49]. In addition to the PPs mentioned above, gingerol, caffeic acid, and quercetin can suppress severe inflammation [42,50,51]. Rodríguez-Ramiro et al. reported that cocoa enriched with PPs and its bioactive properties could reduce severe inflammation effects such as intestinal inflammation and avoid associated cancer [52]. In growing medical research on inflammation, it is suggested that PPs play a potent role in treating inflammation problems. Thus, careful investigation of these substances, either from natural resources or biosynthetic routes, will provide new ways for inflammation research originating from various diseases and ultimately improve public health.

Generally, PPs can modulate inflammation and immunity through various pathways involving NOD-like receptors, Toll-like receptors, NF-κB, inducible enzymes, proinflammatory chemokines, cytokines, and adhesion molecules [53]. Several in vitro and in vivo studies have reported the health-promoting effects of flavanol-rich foods (mainly green tea, cocoa, or grape seeds) and isolated flavanols (epigallocatechin gallate, epicatechin, procyanidins) play an important role in the protection against obesity, diabetes, and MtS. They can modulate the immune system, inflammatory status, and gut microbiota due to the presence of 4-5 hydroxyl groups in their molecules, which provide them with prominent antioxidant potential [53].

#### 2.1.2. Clinical Studies

A recent meta-analysis of random clinical trials demonstrated the effective role of PPs on oxidative stress and inflammation. The findings of this meta-analysis described the potential role of PPs in increasing tacrolimus (TAC) levels [54]

### 2.2. Insulin Resistance/Hyperglycemia

Insulin resistance is the most common MtS in which body cells (fat, liver, and respective muscles) cannot provide an effective response to insulin and cannot consume glucose from the blood to produce energy [53]. Insulin resistance leads to the destruction of insulin-producing pancreatic *B*-cells.

This MtS can further complicate the health problems such as a rise in blood pressure, obesity, unbalanced cholesterol levels, and diabetes problems [55]. This type of syndrome can easily be identified by regular glucose checkups and other corresponding analyses to check the glucose tolerance level in the human body. The origin of this MtS may come from various sources such as the family history of the disease, dietary habits, smoking, age, and others [56]. In addition to a high blood sugar level, diabetes mellitus often has manifestations of metabolic disorders such as obesity, dyslipidemia, and hypertension, which increase the risk of cardiovascular and cerebrovascular diseases and increase mortality [57].

#### 2.2.1. Pre-Clinical Studies

Due to the anti-inflammatory and other favorable medical features of PPs, these compounds have been widely studied for metabolic disorders, such as insulin resistance and associated diabetes problems [58,59]. In this concern, several reports have been done to find favorable use of PPs extracted from various resources to treat insulin resistance [60,61]. Anhe et al. reported that polyphenolic-rich extracts of cranberry could successfully improve insulin resistance and other health problems, including obesity and inflammation in the animal and human body [62]. In various in vivo and in vitro studies on different animals, it is observed that PPs present in green tea can modulate sensitivity towards insulin due to the presence of epigallocatechin, which is known as one of the best health-improving markers [63].

#### 2.2.2. Clinical Studies

Perez et al. investigated the effect of *Aloe Vera* extract enriched with PPs on insulin resistance [64]. The analysis was based on body weight, dietary intake, and plasma composition. Based on the achieved results, they suggested that *Aloe Vera* extract can be an effective strategy to control insulin resistance. However, quantitative analysis of this extract is still needed to introduce this strategy at the commercial medical level. In a study by Hokayem et al., a mixture of PPs from grapes can effectively help to avoid fructose-based oxidative stress and further risk of insulin resistance [65]. In 2008, Anderson confirmed chromium and PPs extracted from cinnamon’s effective role in improving insulin sensitivity. According to his clinical trials, the subjects with the aqueous intake of cinnamon showed excellent improvement in metabolic disorders such as glucose tolerance and insulin resistance [66].

Based on the literature, we can predict that the ongoing interest in PPs from different natural resources could further induce these compounds as stepping pillars of special diets for various diseases. Therefore, consuming PP-containing food is considered an important supplement in antidiabetic therapy [1]. Shahwan et al. found that PPs such as quercetin, resveratrol, and epigallocatechin-3-gallate enhanced glucose uptake in the adipocytes and muscles in type 2 diabetes by activating the AMP-activated protein kinase pathway [67]. Resveratrol, a natural polyphenol detected in more than 70 plants in large doses (≥1000 mg), significantly reduced blood glucose levels [57]. Generally, phenolic compounds can reduce oxidative stress and protein glycation, inhibit the activity of dipeptidyl peptidase and other enzymes related to carbohydrate metabolism, improve pancreatic β-cell functions and increase insulin secretion [68].

### 2.3. Obesity

Obesity, or a body having a higher weight than normal (as per BMI index), is one of the major problems in the new generation and society [69]. Over 1/3 of the world’s population is obese [1]. Obesity is closely linked with several health disorders, such as adipocyte hypertrophy, insulin resistance, diabetes mellitus, systematic inflammation, non-alcoholic fatty liver disorder, coronary heart diseases, cardiovascular diseases, and cancer [20,70,71].

#### 2.3.1. Pre-Clinical Studies

The potential use of PPs by in vivo studies suggested favorable results for obesity if these compounds are introduced in cell cultures. This problem is also mostly associated with MtS and causes further health problems such as diabetes and other intestinal problems. Therefore, it is necessary to maintain the weight measurement according to the respective BMI for a normal body. For weight loss, there is a need to burn extra calories stored in the form of body fat [72]. This can be done either by natural fat-burning compounds or by taking an external supplement that can effectively modulate fat burning. In natural fat-reducing compounds, PPs are known as the best fat burners that induce weight loss by only carefully adding these compounds to the respective diet [73]. As already mentioned, these substances can naturally be found in fruits such as apples, pears, and other green leaves.

#### 2.3.2. Clinical Studies

As oxidative stress and inflammation play a key role in the etiology of obesity, the healing effect of phenolic compounds due to their antioxidant and anti-inflammatory properties was proven in experimental studies [70]. PPs are associated with every type of MtS, from simple fat burn to a healthy heart. Dulloo et al. reported that treating epigallocatechin gallate (90 mg) with a small amount of caffeine could increase energy consumption, ultimately resulting in weight loss due to a higher rate of fat oxidation [74]. Guo et al. reported that the intake of PPs can effectively reverse obesity and other metabolic disorders [75]. They suggested that the long-term intake of a PP-enriched dietary plan is one of the best strategies to lose weight, ultimately offering effective protection from diseases such as heart attack.

As was noticed in the middle of the 20th century, the Mediterranean diet can prevent some chronic diseases related to consuming food, such as coronary heart disease, obesity, etc. [76]. Polyphenol-rich diets, including the Mediterranean ones, which foster the usage of a variety of polyphenol-containing products, could be an effective dietary means of improving the health of patients with MtS [36,77]. Blueberries are PP-rich fruits (flavonoids and organic and phenolic acids), even though the *Vaccinium* plant has been the subject of continuous research to provide a modulated function for obesity. Other PPs such as curcumin and resveratrol have also been reported for anti-obesity to avoid associated metabolic disorders [78]. Considering the anti-obesity features of PPs, introducing these compounds in the dietary plan will provide new medical research to deal with various diseases. As is known, seaweed has been consumed for centuries as a source of valuable bioactive compounds [79]. Their PPs can transform white adipose tissue into “brown” and enhance energy consumption [80].

However, more clinical trials are required to confirm the capability of PPs in the weight loss process and quantify their intake of dietary meals [81]. It is also important to consider that long-term and high dosages of PPs can adversely affect human health. Thus far, there is no exact clue for the safe consumption of these compounds to treat obesity and other metabolic disorders [82]. As obesity is a low-grade chronic inflammation causing insulin resistance, PPs can reduce the risk of type 2 diabetes by reducing obesity [83].

### 2.4. Liver Intoxication

The liver is one of the vital internal organs of the body responsible for regulating more than 500 functions occurring in the human body. The liver’s most important function is to detoxify and neutralize the toxins coming into the body to avoid further health complications [84]. When it comes to liver cleansing, there are several home or market-wide methods available. However, many of these methods are not even tested on a clinical basis or are not regularized by the national drug authorities [85]. Liver detoxification is also associated with the metabolic system.

#### 2.4.1. Pre-Clinical Studies

Many studies have been reported to analyze the effect of dietary intake on the detoxification of the metabolic and liver system. Various reports suggest that dietary nutrients can effectively modulate the metabolic system to detoxify toxins in living cells [86,87]. Different toxins in the human body can lead to various chronic diseases such as diabetes, obesity, and heart problems. Therefore, the dietary intake of the corresponding host may have a key role in preventing toxins [88,89]. A wide range of flavonoids showed promising therapeutic effects in liver damage caused by various toxins. Baicalin, which is found in the herbal drug *Scutellariae radix*, a well-known flavonoid, can effectively alleviate liver damage, mainly by suppressing the signaling pathway and its downstream effectors of inflammatory responses [90].

The PP-enriched fraction from the leaves of *Microcos paniculata* exhibited a significant hepatoprotective effect. It was found that the fraction demonstrated strong free radical scavenging activities and a hepatoprotective impact due to the dual regulation of ROS/mitogen-activated protein kinase (MAPKs)/apoptosis axis and Nrf2-mediated antioxidant response [91]. Curcumin from the *Curcuma longa* rhizome can inhibit inflammation and apoptosis signaling to treat endotoxemia-induced liver failure.

The compound improved levels of antioxidant enzymes and inhibited the activation of the MAPK/NH2-terminal kinase c-Jun cascade in rat liver. In addition, curcumin reduces serum cytokines such as IL-6, IL-1 β, and tumor necrosis factor-α. It improves liver apoptosis by suppressing the phosphatidylinositol-3-kinase/protein kinase B signaling pathway (PI3K/AKT) and inhibition of expression of the cyclic AMP-element binding protein (CREB)/caspase [92]. Another study showed that a polyphenolic extract of *Hibiscus sabdariffa* L. could improve liver steatosis and have a hepatoprotective effect by reducing mitochondrial dysfunction [93]. Silymarin, a mixture of flavonolignans obtained from *Silybum marianum* Gaertneri, has been demonstrated to reduce liver damage and inflammation caused by bisphenol A [94,95]. Resveratrol, a naturally occurring polyphenol with multiple pharmacological properties, including anticancer, antioxidant, antidiabetic, antinociceptive, and antiasthmatic activities, has significant hepatoprotective effects on liver damage. Resveratrol’s mechanisms are associated with inflammation inhibition, enhancing the apoptosis of necrotic hepatocytes, and suppressing oxidative stress [96]. The geraniin and amariin, two ellagitannins from *Phyllanthus amarus,* have demonstrated remarkable properties to protect the mouse liver from alcoholic cytotoxicity by reducing antioxidant enzymes, inhibiting lipid and protein oxidation, stopping the formation of 8-hydroxy-2-deoxyguanosine and modulating Bcl-2-associated X/Bcl2 against apoptosis [97]. The hepatoprotective effect of polyphenolic extract from *Trigonella foenum graecum* (*Fabaceae*) seeds against experimentally induced ethanol-induced hepatic injury and apoptosis was comparable with those of silymarin as a known hepatoprotective agent [98]. Lim et al. established the antioxidant properties of *Amomum cardamomum* L. (*Zingiberaceae*) ethanol extracts in doses of 100 and 200 mg/kg in vivo against CCl_4_-induced acute liver damage using HepG2 hepatocarcinoma cells [99]. Serum levels of key enzymes (glutamic oxaloacetic transaminase, glutamic pyruvic transaminase, and alkaline phosphatase) were significantly increased after using the ethyl acetate fraction of the extract [99]. Chlorogenic acid was a promising therapeutic agent to prevent drug-induced acute hepatotoxicity due to acetaminophen overdose [100]. Hussain et al. revealed the positive effects of herbal complexes enriched with hydroxycinnamic acids from *Cinnamomum cassia* and cinnamic acid on non-alcoholic liver diseases in the induced obesity mouse model [101].

#### 2.4.2. Clinical Studies

There is an urgent need for special clinical trials based on the selection of diet plans to achieve maximum benefits during the liver detox process. In this concern, PPs have proved their capability to detoxify the liver and corresponding metabolic system effectively. The abundance of PPs in nature makes their role more prominent in medical research and has attracted much attention from health care units [102]. In polyphenolic compounds, flavonoids are the most abundant ones. They are usually found in herbs, tea, soy, and vegetables and possess several favorable health features, such as detoxifying various enzymatic activities in the metabolic system [103]. Detoxifying and oxidative stress preventive abilities of flavonoids might be explained by their impact on mitochondria-ER stress-proteasome, as well as autophagy and apoptosis pathways [104]. However, the chemopreventive features of PPs largely depend on their intake and bioavailability among selected subjects. In this regard, many naturally occurring PPs such as anthocyanins (from grapes), flavonols (from dietary plants) [105,106], flavanones (from oranges) [107], and isoflavones (from soybean) [108] have been studied to detoxify various toxins present in the human body effectively. PPs from other herbal sources such as red wine, green tea [109], and black rice [110] is also a good source of detoxification for the liver as these compounds perform antioxidant properties effectively in the living system. It has been shown that the consumption of orange juice, containing a large number of flavonoids, lowers the level of total cholesterol and increases liver cells’ antioxidant capacity compared with the control group [111]. PPs have been shown to have various pharmacological effects on oxidative stress, insulin resistance, lipid metabolism, and inflammation, which are the most important pathological processes in the etiology of liver disease [112,113].

In studies on human hepatoblastoma cancer cells, Buffalo rat liver cells (BRL-3A,) and human liver cancer cells (HL-7702), it was found that isoorientin (also known as homoorientin) has a marked hepatoprotective effect, which can be mediated by complexes of the respiratory chain and the activity of detoxifying phase II enzymes [114].

The above studies with excellent healthy features of PPs suggest that a careful and quantitative intake of these compounds must be accomplished in the human diet to detoxify the metabolic system naturally.

### 2.5. Aging

The idea of extension in life and a beautiful appearance is necessary for every human body to reverse the effects of aging. This effect can be achieved in several ways, either by using different medicines and supplements or by the dramatic physical changes in the body. Thus far, no such dramatic changes have been clinically observed that can prove effective for the subject. In this concern, various remedies are available over the counter, such as home remedies or allopathic medicines. In the medical industry, antiaging products and their corresponding cosmetic sector have promising contributions within the industry [115]. The consumption of these products is in demand worldwide. Consumers continually desire not only the long-term effects of these agents but also require a response after application to the target. Many cosmetic industries have introduced soft focus and lifting effect concepts considering consumer demand.

In the first one, results are observed in long-term applications, while in the lifting effect, an immediate effect of the proposed cosmetics is offered [116]. In various cosmetics, many naturally occurring compounds are used as skin mediators and healing agents. In these naturally occurring compounds, phenolic-based compounds have a very promising demand as antiaging and for other skin diseases such as skin cancer [117,118]. The antioxidative features of phenolic compounds enable extensive usage of these substances in the cosmetic industry as antiaging agents. Many phenolic compounds have been extracted from various sources, such as potato peels, apples, papaya, rosemary, *Crataegus* spp., and *Ginkgo biloba,* and their applications have been investigated for antiaging purposes [119].

Aging is associated with an increased risk of developing diabetes mellitus, neurodegeneration, cardiovascular diseases, osteoarthritis, or cancer [120]. Natural antioxidants might prevent aging-related pathologies via different signaling systems involving ROS and nitrogen species scavenging, with the Nrf2/Keap1-ARE system and the pathway mTOR/Foxo/SIRT1 [121]. However, to enhance the antiaging effect of PPs, various intermediates (ethanol and weak acids) are used to achieve maximum benefits [122]. In the cosmetic industry, the most used PPs are anthocyanins, phenolic acids, and flavonoids because of their excellent antioxidant activity. Anthocyanins are mostly found in colored vegetables and fruits, while flavonoids and phenolic acids are mostly found in various seed sources. Although the use of these compounds is much broader, their extraction and separation are key challenges in commercializing these compounds in medical research [123,124].

#### 2.5.1. Pre-Clinical

Recently, Papaevgeniou and Chondrogianni demonstrated the medical properties of PPs on *Caenorhabditis elegans* (a multicellular model organism) that can ultimately provide better health by favoring the metabolism process [125]. In a study, the use of PPs from *Rosa rugosa* tea for antiaging properties was confirmed by Zhang et al. [126]. They suggested that the presence of 23 different phenolic compounds in *Rosa rugosa* flower showed excellent antioxidant properties to avoid DNA damage and possess combat action towards aging. From the inherent anti-inflammatory and antioxidative properties of PPs, we can conclude that PPs play a vital role in the cosmetic industry. Further investigation into their extraction and separation can provide a promising future for these compounds.

#### 2.5.2. Clinical Studies

For years, the beneficial antioxidant and anti-aging effects of dietary PPs including curcumin, resveratrol, epigallocatechin-3-gallate, oleuropein, punicalagin, myricetin as well as ferulic and rosmarinic acids have been reported [27]. Yamagata and Yamori concluded that isoflavones from soybean as phytoestrogens exert beneficial effects on MtS, increasing the risk of death due to the progression of arteriosclerosis [127]. Natural PPs such as resveratrol, quercetin, epigallocatechin gallate, and apigenin have advantages in healthy aging compared to synthetic drugs for inducing autophagy due to their intrinsic safety and bio-compatibility [120]. Generally, many flavonoids and other PPs can promote health and longevity [128].

### 2.6. Carcinogenesis

Carcinogenesis or oncogenesis is the formation of various cancer types involving a multistep and complex process of normal cell division to malignant ones (cancer cells). Different biological alterations generally identify these processes at genetic, internal cellular, and epigenetic levels. These further cause cell division in dead cells and can occur in almost all body tissues under several circumstances [129]. The general theory of carcinogenesis suggests that DNA mutation is a major cause of developing cancer cells. However, only a few mutations can cause cancer cells while others cannot [130]. Carcinogenesis occurs due to human exposure to carcinogens, which can be chemical, biological, radiative, or viral substances. During the process of carcinogenesis, it is observed that the imbalance response of cytokines and their growth in the normal human body also plays a key role in the formation of cancer cells and their further progress by altering the cell cycle proteins [131]. The anticancer effects of natural phytochemicals, such as PPs, relevant to the modulation of cytokine signaling pathways in various cancer cells are evident from many reports.

#### 2.6.1. Pre-Clinical Studies

Various types of PPs have been reported to suppress the carcinogenic process occurring in the human body [132]. Green tea is a popular useful beverage consumed worldwide, especially in Asian countries (Pakistan, India, China, Japan, and others); it is a source of rich phenolic compounds [133]. The value of green tea is that it provides catechins to the body, which are compounds that contain phenolic rings and a large number of OH groups, which gives them high antioxidant activity. Epigallocatechin-3-gallate is of great importance in providing the medicinal properties of green tea. Epigallocatechin 3-gallate, a polyphenolic component from green tea infusion, possesses anti-inflammatory and anti-cancer properties [134]. In different carcinogenesis models—chemical, genetic, UV, and irradiation—a significant anti-carcinogenic effect of green tea extract has been established [135,136].

Yang et al. reported the effective capability of tea-based PPs, such as tea catechins, to suppress carcinogenesis and inflammation processes in the human body [137]. However, the mechanism by which polyphenol disrupts undesirable biological alterations is still unclear. The use of tea catechins to treat carcinogenesis in animals and humans has been studied and employed successfully. Many researchers have reported the mechanism of red wine phenols for carcinogenesis suppression, such as tumors in mice and colon cancer in rats [138]. The fruits of feijoa (*Feijoa sellowiana* Berg.) also contain a large number of bioactive compounds, including PPs, which have anti-tumor activity [139]. Glycycoumarin, isolated from licorice, exhibits antitumor activity by directly affecting the protein kinase (TOPK) of the oncogenic kinase T-LAK derived from cells [140]. In experiments in vitro and in vivo, it was found that curcumin modulates the activity of many signaling pathways, affects the cell cycle, induces apoptosis, enhances the detoxification of several carcinogens, and prevents the development of several tumors [141].

Chemopreventive effects of PPs include anthocyanins, epigallocatechin gallate, ellagitannins, punicalagin, quercetin, resveratrol, and theaflavin for the treatment of melanomas mediated by several signaling pathways against skin carcinogenesis and metastasis [142]. Matsuno et al. revealed that resveratrol and related compounds could improve genome stability in mouse embryonic fibroblasts by protecting the cells against the induction of mutations [143]. It could contribute to the suppression of cancers caused by genomic instability. The anti-inflammatory effects of the flavonol quercetin on colon carcinogenesis were studied after inducing by azoxymethane/dextran sodium sulfate in mice [144]. Quercetin also significantly reduced the number and size of colon tumors. Oral administration of rosmarinic acid (100 mg/kg body weight) to experimental hamsters with 7,12-dimethylbenz(a)anthracene-induced oral carcinogenesis completely prevented tumor formation. The authors suggested that rosmarinic acid suppresses oral carcinogenesis by improving the status of lipid peroxidation and antioxidants and stimulating the activity of detoxification enzymes [145]. This natural polyphenol dose-dependently inhibited the cell growth and propagation of human oral cancer cells [146]. Rosmarinic acid is very specific for the plants of the *Nepetoideae* Burnett. Sa subfamily of the *Lamiaceae* Martinov family [147,148]. Rao and co-authors considered that an appropriate diet is an effective approach to preventing cancer and lifestyle-associated diseases; pigmented varieties of cereals (rice, barley, oats, and sorghum) containing anthocyanins, protocatechualdehyde, etc., were discussed as important chemopreventive agents [149].

#### 2.6.2. Clinical Studies

Many clinical trials have demonstrated that tea PPs can prevent and treat colorectal cancer developed through different mechanisms such as the impact of genomic abnormalities, oxidation stress, dysbiosis, and diet. Tea PPs inhibit the growth of colorectal cancer by modulating the gut microbiota and improving the immune system [150]. Several PPs extracted from various sources, such as fruits, beans, cocoa, vegetables, and olives, have been studied for skin cancer [151]. Generally, PPs suppress DNA damage by inhibiting ROS formation that ultimately terminates cancer cell production, activating survival or autophagic and pro-apoptosis mechanisms [152]. Recently, it has been suggested that phytochemicals, including PPs, can provide an effective approach to treating cancer caused by UV rays [153]. The extract of these phytochemicals can be included in cosmetics to avoid skin problems and counteract carcinogenesis. Grapes are also one of the best sources that contain a rich amount of PPs that can effectively take part in combating carcinogenesis [132]. Lower morbidity due to cancer in Japan and China, compared with the European countries and the USA, might be explained, in particular, by the traditional diet of these countries, containing high contents of soy products. Soy is rich in isoflavones, which have estrogen-like activity (phytoestrogens) and render both estrogenic and antiestrogenic effects. The main isoflavone of soy is genistein, a polyphenol with a large number of hydroxyl groups which causes its high antioxidant activity. Genistein is a promising natural substance for preventing hormone-dependent tumors [154,155].

Curcumin, obtained from the roots of the *Curcuma longa,* also causes significant research interest [156]. Curcumin is used as a part of traditional Indian cousin. The presence in the molecule of two phenolic rings with hydroxyl groups and a large number of double bonds can contribute to the powerful antioxidant properties of curcumin.

### 2.7. Cardiovascular Diseases

The past few years have witnessed an extreme transformation in habits, pushing modern populations away from a natural diet and toward unhealthy foods and a sedentary lifestyle. The risk of developing CVDs has increased due to the integration of the modern lifestyle with a persistent intake of harmful intoxicants, including tobacco and misuse of drugs [157].

CVDs are a group of several heart-related complications, such as hypertension, heart failure, hyperlipidemia, acute coronary syndrome, peripheral artery disease, and stroke. Inflammation, atherosclerosis, immune responses, and any physical damage are the most common causes linked to the pathogenesis of heart stroke and failure. Furthermore, increased ROS generation results in altered molecular pathways and endothelial dysfunction; both play a significant role in the etiology of CVDs [158]. Several chemical-based drugs, such as antiplatelet drugs and cholesterol-lowering agents, are extensively utilized for CVDs treatment. However, these drugs pose several harmful effects.

Consequently, the use of herbal products is expanding rapidly in the 21st century [159]. Most plant species have remarkable safety records, making them a unique candidate for treating heart-related diseases [160]. PPs are the most promising plant compounds which showed significant cardioprotective properties due to their antioxidant potential. Recently, several research studies have evaluated their anti-atherosclerotic and immunomodulatory properties through pre-clinical and clinical trials. These studies highlight the importance of polyphenolic compounds as a natural way of reducing the risk of developing CVDs [161,162,163].

#### 2.7.1. Pre-Clinical Studies

Kleemann et al. conducted in vitro (on cultured HuH7 hepatoma cells) and in vivo (2 mice models: humanized inflammation model and humanized atherosclerosis model) studies to check the anti-inflammatory role of quercetin on CVDs [164]. The results of this research indicated positive effects on inflammatory risk factors such as human C-reactive (CRP) and fibrinogen proteins, mitigating atherosclerosis and, ultimately, CVDs. Luo et al. recently demonstrated that by lowering the levels of p47phox and NADPH-related free radical formation, quercetin greatly reduced endothelial dysfunction and atherosclerosis, which is a key role for polyphenols in heart diseases [165]. Another study showed that pretreatment of the induced cardiomyocyte damage model reduces oxidative stress and improves cell viability and cardiomyocyte damage [166].

Naringin, another important polyphenol, also showed significant potential cardioprotective effects. According to an in vivo study, naringin treatment in mice decreased mitochondrial tricarboxylic acid enzyme activity at different doses, i.e., 10, 20, and 40 mg [167]. Naringin’s cardioprotective role is also linked to its potent ability to block the ROS-activated MAPK signaling pathway linked to hyperglycemia [168,169,170]. In the pulmonary thromboembolism-induced pulmonary hypertension model, resveratrol (2 doses: 10 mg/kg) down-regulated the monocyte chemoattractant protein-1 (MCP-1) and P-p38MAPK and thus substantially decreased baseline arterial blood pressure [171]. In most preclinical investigations, resveratrol improved diastolic pressure, cardiovascular remodeling, cardiac function, and systemic blood circulation. It prevented heart fibrosis in mice and rats with stress-overburden-induced heart failure models [172].

The anti-atherosclerosis effects of curcumin were studied in the ApoE^−/−^/LDLR^−/−^ mice model by administration of 0.3 mg per day. The results of this study revealed that curcumin prevented the development of atherosclerosis even if it had no exceptional therapeutic efficacy on mice’s body mass and serum lipids [173]. Both *in vitro* and in vivo studies demonstrated the therapeutic action of salvianolic acid for CVDs [174,175,176]. Compared to control, catechin-rich water (50 g per day) was given to 40 ApoE defective mice for six weeks, resulting in a 39% decrease in atherosclerosis. LDL levels dropped by 31%, and oxidation susceptibility decreased [177]. Administration of 10 and 15 mg/kg of caffeic acid significantly boosted nitric oxide absorption, improved catalase activity, decreased glutathione, and reduced malondialdehyde concentration in rats [178].

#### 2.7.2. Clinical Studies

Several clinical studies have also been conducted to evaluate the effective role of PPs on CVDs. A pilot study conducted by Mankowski et al. examined the activity of resveratrol at higher doses on cardiovascular risk biomarkers in obese geriatrics subjects. Results of a pilot study involving obese geriatrics patients revealed that a higher dose of resveratrol (300 mg), when compared to placebo, substantially increased both total plasminogen activator inhibitor (tPAI) and circulating vascular cell adhesion molecule-1 (sVCAM-1). Higher doses of resveratrol were also associated with an increase in other biomarkers, including oxidized low-density lipoprotein (oxLDL), soluble ICAM-1, and soluble E-selectin (sE-selectin) [179]. In a cohort trial with 5115 participants over 20 years, Reis et al. addressed the remarkable effectiveness of green tea for coronary heart disease [180]. Additionally, large-scale cancer nutrition cohort prevention research findings indicate that consuming polyphenolic substances, particularly flavan-3-ols, might effectively lower the risk of CVDs [181,182].

Several observational and experimental studies have demonstrated the link between consuming a diet high in polyphenols and the decreased risk of developing heart illnesses [183]. Results of a multicentered randomized clinical trial reported that consuming a polyphenol-rich diet for five years reduced the death rate in CVD patients by 37% and improved the survival rate [184]. Similarly, cohort clinical research, including an elderly population (>65 years old), revealed that long-term consumption of polyphenols enhances the average life span of old cardiac patients [184]. Stilbenes and lignans are reported to be the most efficient among all polyphenols, which decreased the overall death rate [7].

### 2.8. Other Health Problems

In the fourth century B.C., Hippocrates emphasized the importance of diet in health and disease, saying, “death sits in the bowels” [185]. Many in vivo studies and human trials suggest that gut microbiota dysbiosis is involved in gastrointestinal diseases and obesity, diabetes, and other MtS [35]. Recent data have revealed the ability of foods rich in polyphenols to modulate intestinal dysbiosis present in various diseases by reducing the number of potential pathobionts [35].

#### 2.8.1. Pre-Clinical Studies

Bouyahya et al. analyzed numerous in vivo and clinical trials [44]. They concluded that berry-derived PPs possess significant therapeutic properties and alleviate diseases related to abnormal gut microbiota, including inflammation and colon cancer. Shabbir et al. found that curcumin, quercetin, and catechins could improve gut health and help alleviate the rate of MtS [186]. Duarte et al. have ascertained that gut microbiota in the presence of PPs plays a fundamental role in controlling obesity, possibly through brown adipose tissue activation [70].

As is known, hyperlipidemia is a risk factor for the development of CVDs [187]. Atherogenic dyslipidemia associated with a pro-inflammatory state in MtS could increase the risk of fatty liver, neurodegenerative diseases, and even certain types of cancers [188]. PPs can cause/lipoprotein metabolism in humans. It was found that rosmarinic acid can significantly decrease total plasma cholesterol and triglytriglycin levels as well as body weight in fat-fed mice [187]. Numerous in vivo and in vitro studies demonstrated that apigenin and other PPs were effective against metabolic disease due to their antioxidant and anti-inflammatory properties [189,190].

Such PPs as flavonoids, stilbenes, phenolic acids, and methylxanthines from cocoa beans are known to be mostly responsible for the health benefits of dark chocolate, in particular preventing neurodegenerative and cardiovascular diseases [191]. Flavonoid compounds were suggested as the best PPs due to their several healing effects synergetic with stem cells in treating neurodegenerative diseases [192]. Phlorotannins from brown seaweeds are promising natural compounds to tackle neurodegeneration [193,194]. Some kinds of tea can reduce fat accumulation in experimental animals by increasing lipid metabolism [195].

#### 2.8.2. Clinical Studies

Pourhabibi-Zarandi et al. found that curcumin supplementation (500 mg once a day for eight weeks) improved such metabolic parameters as glycemic index, lipid profile, inflammatory factors, and obesity values in women with a diagnosis of rheumatoid arthritis [196].

Table 1 summarizes the current state of the Mts treatments with PPs from clinical studies.

## 3. Proposed Panel of Polyphenols

It should be mentioned that PPs possess the ability to facilitate the MtS at different levels (Table 2). Several subclasses of flavonoids have proven free-radical scavenging activity and another valuable therapeutic potential: catechins (e.g., gallocatechin, epicatechin, epigallocatechin), flavones (e.g., luteolin, apigenin), flavanones (e.g., hesperidin, naringenin), anthocyanidins (e.g., cyanidin, delphinidin, pelargonidin), flavonols (e.g., quercetin, rutin, myricetin), and isoflavones (e.g., genistein) [198]. For instance, anthocyanins are water-soluble pigments that exist mainly in glycosylated forms. They are responsible for fruits and vegetables in red, purple, and blue colors [199]. The glycoside forms of cyanidin, delphinidin, pelargonidin, malvidin, peonidin, and petunidin are the major anthocyanins in plants [44,199].

Berry-derived PPs are mainly obtained from the fleshy fruits of strawberry, blueberry, mulberry, currant, raspberry, blackberry, barberry, rosehip, and gooseberry [44]. Berries are rich sources of pigments, particularly anthocyanins (up to 5 g/kg), which are responsible for their red, blue, or purple colors. They also contain flavonols, phenolic acids, tannins, etc. Due to their ability to cross the blood-brain barrier, PPs with low molecular weight have beneficial antioxidant effects.

**Figure 2:**
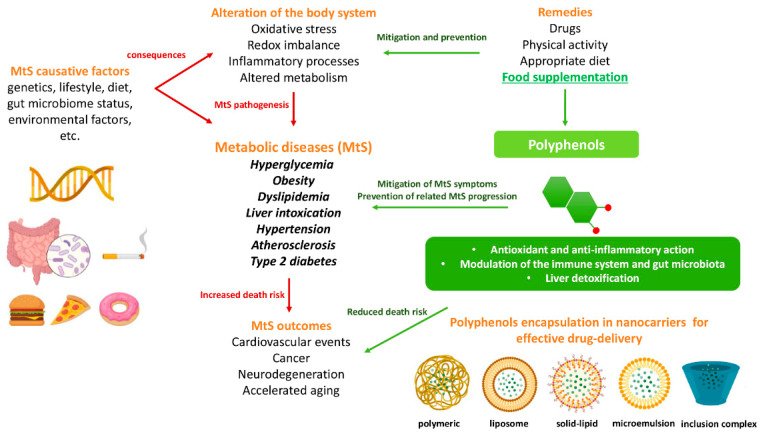
Influences of PPs on different manifestations of MtS.

**Figure 3:**
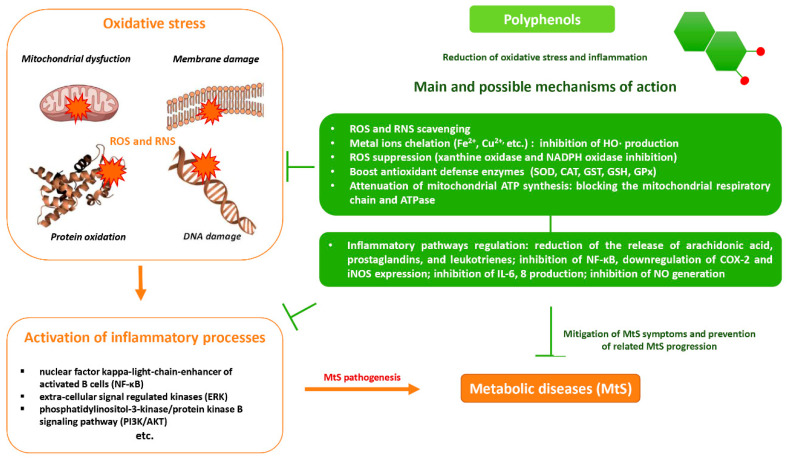
Overall mechanisms of action of PPs against oxidative stress and inflammation leading to MtS.

The potential benefits of PPs on human health make them the best micronutrients obtained from plant foods. These active principles possess excellent antioxidant and anti-inflammatory properties that ultimately favor the metabolic system of the human body and can avoid various chronic diseases (Figure 2 and Figure 3). Epidemiologic and related case studies on mouse and human diets demonstrate that daily intake of PPs can easily prevent and provide efficient treatment for severe metabolic diseases that may cause complicated health conditions.

It could be concluded that a single source of PPs, not always and not everywhere, can fulfill an effective role. Generally, the benefits of PPs involve the mechanism of bioactive scavenger theory in which free radicals are potentially absorbed by these healthy compounds and converted into a stabilized complex [222].

Recent studies suggested that various phenolic compounds can be made artificially or with the help of natural supplements that can influence and terminate the growth of pathogens in the gut. Thus, a careful investigation is required to provide mechanistic studies of various phenolic compounds.

## 4. Effective Delivery of Polyphenols to the Target

People are particularly interested in dietary PPs as there is a growing belief that their constant intake is healthy for the body. However, before reaching the target tissues, ingested PPs are substantially degraded in the digestive system or other sites, reducing their beneficial effects. Furthermore, some PPs are photosensitive and rapidly oxidized into undesirable forms. Although polyphenolic substances have been found to have several benefits, their pharmaceutical use in humans is currently restricted due to these limitations [223,224,225,226]. In recent years, several attempts have been made for intact distribution or delivery of PPs to target organs and tissues. Conversion into inactive forms by microbes, pH, enzymes, and the blood-brain barrier are the most common obstacles encountered by PPs before reaching the target organ. To overcome these challenges, several delivery systems have been introduced. Nanoparticles, liposomes, and microemulsions are the major delivery systems for their effective biodistribution (Figure 2) [227,228]. The most investigated biodegradable and biocompatible polymeric materials that enclose polyphenolic compounds are nanomaterials. Nanoshells, nanocarriers, solid lipid nanoparticles, cyclodextrins, liposomes, and micelles are the most commonly used nanoparticle-mediated delivery systems for PPs [229,230]. There are several methods for delivering these nanoparticles: orally, intravenously, intraperitoneally, and transdermally. Due to membrane adhesion and permeability properties, nanocarriers can transport relatively high concentrations of PPs to the intestine, effectively maintain their integrity, and increase their effectiveness [231].

In an in vitro study, Mathew and colleagues showed that curcumin nanoparticles enclosed in PLGA (poly lactic-co-glycolic acid) attached to Aβ clusters facilitated their dissociation. This study opened the door to minimize the amyloid-plaque development in Alzheimer’s by delivering curcumin-PLGA nanoparticles across the blood-brain barrier [232]. In a rat model of 3-nitropropionic acid-induced Huntington’s disease, it was observed that curcumin-encapsulated solid lipid nanoparticles effectively reduced mitochondrial dysfunction [233]. A remarkable research study demonstrated that solid-liquid nanoparticles functionalized with the anti-transferrin receptor (OX26) monoclonal antibody offered a reliable carrier to deliver the resveratrol and grape skin extract to target the brain and treat neurodegenerative disease [234]. Oral administration of resveratrol-loaded PLGA nanocarrier showed enhanced resveratrol bioavailability [235]. In another recent study, resveratrol oral bioavailability was enhanced in Sprague Dawley rats by binding the galactose ligand (N-oleoyl-d-galactosamine) on the surface of the resveratrol-loaded PLGA nanoparticles [236].

The literature notably documented that using polyphenol-loaded nanocarriers has increased their antioxidant and anti-inflammatory effects [237,238,239,240,241]. Therefore, although there are several types of delivery systems that have been assessed through in vitro and in vivo studies for improved bioavailability and target delivery of PPs in metabolic disease, very few clinical trials involving humans have been conducted so far to assess the effect of different delivery systems for PP delivery to target tissues and organs in metabolic diseases. Future clinical studies should be conducted.

## 5. Effect of Chemical Structure on Biological Activities of Polyphenols

Chemical structures have a considerable impact on the bioavailability and particularly the absorption of the compounds such as PPs, which in turn greatly influence the potential of biological activities such as antioxidant properties [242,243]. The physicochemical and biological characteristics of the substance may differ significantly even when only one of the factors is slightly altered. Similarly, every polyphenolic compound has a particular structure that greatly impacts its relevant biological activities and properties [244].

**Figure 4:**
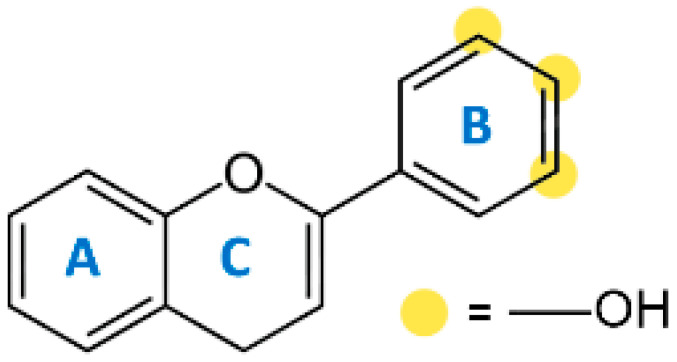
Flavonoid skeleton. The possible positions of the hydroxyl groups in the B-ring are highlighted.

Various mechanisms of radical scavenging activity, including the capacity for metal ion chelation and sequestration, are significantly influenced by the conformation, substitution, and total number of hydroxyl groups of polyphenol compounds [245,246]. The B-ring hydroxyl (Figure 4) is the most important factor in scavenging ROS and RNS since B-ring hydroxyl provides hydrogen and electron to hydroxyl, peroxyl, and peroxynitrite radicals stabilizes these radicals, and generates a rather stable flavonoid radical [247].

The lipid peroxidation is greatly inhibited by a 3′,4′-catechol structure in the B-ring. Because of this property, flavonoids are the most potent antioxidants of peroxyl, superoxide, and peroxynitrite radicals [245]. In vitro studies have displayed epicatechin and rutin as potent antioxidants and lipid peroxidation blockers [248]. An orthosemiquinone radical that is a potent scavenger is produced due to oxidation on the B-ring of PPs bearing catechol groups. Flavones without the catechol system undergo oxidation and produce unstable radicals with little antioxidant capability [249]. According to the research, PPs with an unsaturated 2-3 bond conjugated to a 4-oxo group are effective antioxidants compared to PPs with either one or both properties missing. The resonance action of the aromatic nucleus occurs due to the linkage of A- and B-rings, which gives the polyphenol radical stability [250]. Antioxidant activity is greatly influenced by the presence, arrangement, form, and total quantity of glycosidic bonds in polyphenols [251].

Similarly, the antibacterial potential of polyphenols depends on the formation of a complex between proteins of bacteria through hydrogen and covalent bonding [252,253]. According to a study, the B-ring of PPs may form complexes or establish hydrogen bonds with the nucleic acid bases, which would further prevent bacteria from synthesizing DNA and RNA [254,255]. For a flavanone to have anti-MRSA (methicillin-resistant *Staphylococcus aureus*) action, the A-ring must be 5,7-dihydroxylated, and the B ring must be 2′,4′- or 2′,6′-dihydroxylated [256]. PPs’ structure-function link is the perfect illustration of their main biological functions. A wider collection of chemical compounds with comparable structural characteristics should be the subject of further study to check the interconnectedness of structure and activity. Such research will offer knowledge that can be utilized to develop physiologically significant compounds that can be employed as medicines, antioxidants, or inflammatory agents in the pharmaceutical, food, and medical industries.

## 6. Synergetic Interaction of PPs and Some Challenging Issues for Their Applications

Niewiadomska et al. noted that there is no universal polyphenolic compound for improving all pathological effects [1]. Even though most PPs can improve lipid and glucose profiles, the administration of specific phenolic compounds leads to certain changes. At the same time, combining related PPs may provide synergistic effects and lead to more significant health benefits. It was found that purified phytochemicals isolated from medicinal plants may be less effective than their combination due to the synergistic impact sets of interacting compounds [188].

Medical research on pathogen chemoprevention is paying close attention to PPs for their actual health characteristics at the molecular level. For instance, the chemopreventive compounds may include pharma drugs (tamoxifen) and natural antioxidants such as PPs or a combination of both to provide micronutrients to fight against pathogens in the metabolic system. Rodríguez-Vera et al. highlighted that PPs and stem cell therapies have exhibited improvements in in vitro and in vivo models of metabolic and neuronal diseases [192].

Lewandowska et al. reported the synergetic combinations of various PPs as an anticancer drug in combination with synthetic drugs [257]. Brahmbhatt and coworkers investigated the synergetic interaction of different PPs (gingerols:6,8,10 and shogaol) [258]). The results of this study suggested that a synergetic combination of these PPs can provide excellent chemopreventive measures against various types of cancers. Gundala et al. reported enhanced (100-fold to the parent one) biochemical activity of polyphenolic extracts by using a new fractionation process [259]. Various studies have reported that a synergetic combination of different Chinese medicines containing a combination of phenolic compounds can actively boost the immune system and the digestive system, ultimately favoring metabolic disorder prevention [260,261]. Many other synergetic interactions of various phenolic compounds have been investigated for inflammatory diseases. Morre et al. reported ten-fold better biochemical activity after combining epigallocatechin gallate with epigallocatechin and other phenolic presents in the extract [262].

Furthermore, researchers have also investigated the synergetic interaction of grape extracts with green tea phenolic compounds and reported their mutual extraordinary chemopreventive properties for MtS associated with cancer [263]. Kurin et al. reported an interaction-based study of phenolic compounds from three kinds of red wine and their corresponding mixed extracts [264]. They reported that a synergetic combination of PPs can provide better antioxidant activity than the individual one. In the next study, Kurin et al. reported an effective treatment approach for atherogenesis by interacting with various PPs (quercetin, ethyl gallate, and resveratrol) obtained from grapes [264]. In studies, the interaction of grape-based phenolic with ethanol is also evident in treating inflammation problems [202]. Scheepens et al. reported an extensive review of designed synergetic interactions of PPs to improve their bioavailability and ability to combat various diseases [265]. The designed modification may enhance the bioavailability of various oral PPs. Based on literature studies, it is clear that the synergetic interactions of various PPs play a vital role in chemopreventive measures and need more careful quantitative investigation for its promising future in medical research.

Thus, natural PPs have great antioxidant and anti-inflammatory potential, which broaden their medical applications to treat MtS marked by low-grade chronic inflammation. Despite such advantages as low cost and a vast range of plant resources, some issues of using PPs are still challenging [15]. For instance, the content of PPs in the different plant raw materials varies significantly. Other extraction methods, drug delivery systems, and different bioavailability further limit experimental reproducibility and clinical trials of PPs. Finally, different genetic backgrounds and lifestyles could also lead to various effects of PP intake in humans.

Resveratrol, curcumin, anthocyanins, and many other natural PPs are known for their therapeutic potential in metabolic disorders and possess limited bioavailability [156,186,204,209,266,267]. Therefore, advanced delivery systems such as nano-formulations enhance polyphenols’ clinical potential and therapeutic effectiveness.

The optimal doses and duration of flavanols administration for obtaining a beneficial effect in humans on major metabolic disorders should be further investigated [53]. Generally, the appropriate amounts and delivery methods of PPs should be scheduled for preventing or alleviating MtS [15]. Due to the differences between animal and human models, further clinical trials are needed to elucidate the effects of polyphenolic compounds on MtS patients.

## 7. Conclusions

In growing medical and pharmaceutical research, PPs are one of the best naturally occurring compounds possessing several health benefits and play a vital role as natural chemopreventives of metabolic disorders with accelerating aging, such as elevated blood sugar, obesity, dyslipidemia, hypertension, liver intoxication, colon cancer, and neurodegeneration. The excellent biological activities of PPs and their scavenging features towards free radicals and toxins have provided a new road map to medical research. Thus far, many phenolic compounds have been investigated that can play a key role in preventing and treating various metabolic disorders at their level. However, the synergetic interactions of individual PPs with other phenolic compounds and synthetic drugs are also receiving much attention to improve bioactivities and their corresponding bioavailabilities at oral levels. The literature study reveals that the abundance of PPs possessing different chemical structures and biological fate makes them challenging as a health marker for long-term benefits. There is a dire need for mechanistic studies at clinical levels that can quantify the required dosages of individual PPs and their synergetic combinations. Regular consumption of PPs can help to alleviate many MtS manifestations. Thus, the main focus should be on scientific evidence to provide safe consumption guidelines for these compounds to achieve excellent benefits. Considering various inherent health markers of phenolic compounds, their intake should be included in special diet plans, and further clinical trials could be taken.

## Figures and Tables

**Table 1:** The current state of the Mts treatments with polyphenols from clinical studies.

PPs Type and Main Features of Treatment	Pathologies and Mechanism of Action	Refs.
**Oxidative Stress and Inflammation**		
**Oleuropein, hydroxytyrosol, curcumin, resveratrol, epigallocathechin**	Cell protection (redox homeostasis) through the activation of vitagene signaling pathways	[33,197]
**Grape products containing PPs (resveratrol, proanthocyanidin, quercetin, etc.)**	Significant increase in the levels of total antioxidant capacity and oxygen radical absorbance capacity as well as improving various enzymatic systems such as superoxide dismutase or glutathione peroxidase (dependently on the dosage)	[54]
**Genistein, silymarin caffeic acid, chlorogenic acid, ellagic acid**	Healing chronic inflammation is the key pathomechanism of obesity-related metabolic disorders (insulin resistance, type 2 diabetes, and cardiovascular diseases)	[43]
**PPs from cocoa, fruits, and vegetables**	Alleviating the oxidative damage and inflammation parameters	[6,7,10,83]
**Diabetes**		
***Aloe Vera* extract (enriched with PPs), PPs from grapes,** **and cinnamon**	Control of insulin resistance	[64,65,66]
**Quercetin, resveratrol and epigallocatechin-3-gallate**	Enhancing glucose uptake in the adipocytes and muscles in type 2diabetes by the activation of the AMP-activated protein kinase pathway	[67]
**Resveratrol**	Reducing blood glucose levels	[57]
**PPs from fruits and vegetables**	Protecting pancreatic β-cells and activating glucose/lipid metabolism pathways, affecting glucose absorption and uptake	[67,83]
**Obesity**		
**Epigallocatechin gallate**	Increasing energy consumption and weight loss due to a higher rate of fat oxidation	[74]
**The total PP content (measured in urine samples using the Folin–Ciocalteu method)**	Long-term intake of PPs led to significant loss of weight	[75]
**Curcumin and resveratrol**	Anti-obesity effect to avoid associated metabolic disorders	[78]
**Brown seaweed PPs**	Effective regulation of metabolic disorders via correction of fat function (transforming white adipose tissue into “brown” and enhancing energy consumption)	[80]
**PPs from fruits and vegetables**	Reducing lipid accumulation and regulating intestinal microflora	[83]
**Liver Intoxication**		
**Silymarin/Silybin**	Hepatoprotection, preventing and treatment of chronic liver disease	[85]
**Flavonoids** **(anthocyanins, flavonols, flavanones and isoflavones)**	Detoxifying and oxidative stress preventive abilities of flavonoids through regulation of the autophagy and apoptosis pathways as well as by impact on mitochondria-ER stress-proteasome	[104,105,106,107,108]
**Foods’ PPs (whole-foods approach)**	It affects the activity of detoxification pathways, including Nrf2 signaling, phase I cytochrome P450 enzymes, phase II conjugation enzymes, and metallothionein	[102]
**Aging**		
**Resveratrol**	Vascular dysfunction in aging	[67]
**Resveratrol, quercetin, curcumin and catechins**	Modulation of some of the evolutionarily conserved hallmarks of aging, such as oxidative damage, cell senescence, and autophagy	[117]
**Flavonoids, curcumin and resveratrol**	Disruption of age-associated deterioration in physiological function	[123]
**Isoflavones from soybean**	Anti-arteriosclerotic effect	[127]
**Flavonoids and tannins**	Modulating genes associated with stress defense, drug-metabolizing enzymes, detoxification, and transporter proteins	[188]
**Carcinogenesis**		
**Epigallocatechin and other tea PPs**	Chemopreventive effects on colorectal cancer	[150]
**Pomegranate fruit extract, green tea PPs, grape seed proanthocyanidins, resveratrol, genistein, silymarin, and delphinidin**	Inhibition of photocarcinogenesis (melanoma, squamous cell carcinoma, basal cell carcinoma)	[151,153]
**Isoflavones from soybean**	Prevention of prostate and breast cancer	[154,155]
**Cardiovascular Diseases**		
**Resveratrol**	Increasing total plasminogen activator inhibitor and circulating vascular cell adhesion molecules	[179]
**Green tea PPs**	Prevention the coronary heart disease	[180]
**Cocoa flavanols**	Improving the levels of biomarkers for cardiometabolic disorders	[181,182]
**Lignans, flavonoids, and hydroxybenzoic acids**	Diminishing risk of major cardiovascular disorders (ischemia, myocardial infarction, stroke)	[9]
**Rheumatoid Arthritis**		
**Curcumin**	Improving metabolic parameters and inflammatory factors in women with rheumatoid arthritis	[196]

**Table 2:** The main polyphenols and underlying mechanisms of their pharmacological activity in MtS treatment and prevention.

The Common Name of Polyphenolic Compound	Structural Formula and IUPAC Name	Class of Phenolic Compounds	Main Sources	Main Targets of Action (Metabolic Diseases and States)	Refs.
Resveratrol	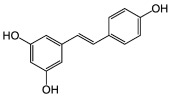 3,5,4’-trihydroxystilbene	Stilbenes	Grapes, raspberries, mulberries, blueberries, apples, plums, and peanuts	- Antioxidant, antidiabetic, anti-obesity, antinociceptive, anticancer, hepatoprotective effects - Modulation of cytokines and suppression of inflammatory disease - Enhancing glucose uptake in the adipocytes and muscles in people with diabetes (by the activation of the amp-activated protein kinase pathway) - Maintenance of genome stability - Autophagy inducers in aging research	[47,48,49] [57] [67] [78] [96] [143] [200] [201] [202]
Curcumin	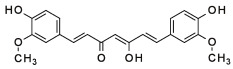 (1*E*,6*E*)-1,7-Bis(4-hydroxy-3-methoxyphenyl)hepta-1,6-diene-3,5-dione	Curcuminoids (diarylheptanoid)	Turmeric (*Curcuma longa*) rhizome	- Antioxidant, anti-inflammatory, anti-obesity, hepatoprotective, anti-atherosclerotic, and anti-diabetic properties - It can effectively suppress inflammatory mediators such as cyclooxygenase - Inhibiting the inflammation and apoptosis signaling for the treatment of endotoxemia (liver failure) - Improving gut health, glycemic index, lipid profile, and obesity values - Treatment of chronic diseases (diabetes, gastrointestinal, neurological, cardiovascular, several types of cancer)	[49] [67] [78] [156] [141] [186] [196] [203] [204]
Quercetin	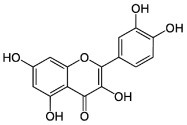 3,3′,4′,5,7-Pentahydroxyflavone	Flavonoids (flavonols)	Fruits and vegetables (mainly of yellow or orange color)	- Capability to suppress oxidative stress and severe inflammation, - Enhancing glucose uptake in the muscles and adipocytes, inducing autophagy - Improving gut health - Suppressing colon carcinogenesis through its anti-inflammatory effects	[42] [61] [105] [106] [120] [144] [186] [202] [205] [206] [207]
Epigallo-catechin gallate	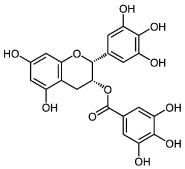 (2*R*,3*R*)-3′,4′,5,5′,7-Pentahydroxyflavan-3-yl 3,4,5-trihydroxybenzoate	Flavonoids (catechins)	Green tea	- Strong antioxidant and anti-inflammatory properties - Modulating sensitivity towards insulin in case of type 2 diabetes - Improving the dyslipidemia state - Anti-obesity influence (stimulating weight loss) - Inhibiting carcinogenesis, inducing autophagy	[53] [63] [67] [120] [134] [135] [136] [186]
Anthocyanins	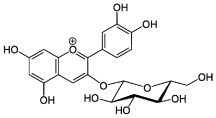 (2S,3R,4S,5S,6R)-2-[2-(3,4-dihydroxyphenyl)-5,7-dihydroxychromenylium-3-yl]oxy-6-(hydroxymethyl)oxane-3,4,5-triol chloride (Cyanidin-3-glucoside)	Flavonoids (anthocyanins)	Berries and flower corollas (in red, blue, or purple colors)	- Management of various metabolic disorders, including diabetes, obesity, high blood pressure, and neurodegeneration - Preventing free radical production - Protecting *β*-cells	[199] [208] [209]
Genistein	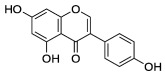 4′,5,7-Trihydroxyisoflavone	Flavonoids (isoflavone)	Mainly *Fabaceae* plants (soy-beans in particular)	- Suppression of free radicals - Inhibition of inflammation - Promotion of apoptosis - Prevention of hormone-dependent tumors through modulation of steroidal hormone receptors	[108] [127] [210]
Naringenin	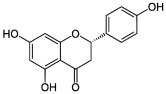 (2*S*)-4′,5,7-Trihydroxyflavan-4-one	Flavonoids (flavanone)	Citrus fruits (oranges, lemons, grapefruits, etc.)	- Strong anti-inflammatory and antioxidant effects, - Treatment of diabetes, obesity, hypertension, and other manifestation of MtS - Improving lipid metabolism	[169] [211]
Apigenin	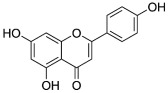 4′,5,7-Trihydroxyflavone	Flavonoids (flavone)	Celery, parsley, *Lamiaceae* plants	- Effectiveness against cardiometabolic diseases due to the antioxidant and anti-inflammatory properties - Inducing autophagy, - Anticancer effect	[120] [189] [212] [213]
Luteolin	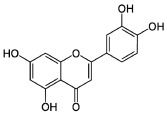 3′,4′,5,7-Tetrahydroxyflavone	Flavonoids (flavone)	Celery, carrot, parsley, broccoli, oranges, chamomile tea, and *Lamiaceae* plants (thyme, oregano, rosemary, etc.)	- Prominent antioxidant and anti-inflammatory effects - Treatment of glycolipid metabolism disorders (in case of obesity and diabetes)	[46] [198] [214]
Silybin	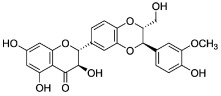 Silybin A (2*R*,3*R*)-3,5,7-Trihydroxy-2-[(2*R*,3*R*)-3-(4-hydroxy-3-methoxyphenyl)-2-(hydroxymethyl)-2,3-dihydro-1,4-benzodioxin-6-yl]-2,3-dihydro-4H-chromen-4-one	Flavonolignan (silymarin group)	Milk thistle (*Silybum marianum*) fruits. Silymarin is a flavonoid mixture in which silybin is the major one.	- Antioxidative, anti-inflammatory, antiapoptotic, hepatoprotective properties, - Preventing and treatment of chronic liver disease	[43] [94] [95]
Phlorotannins	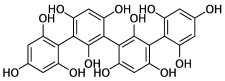 Tetrafucol A, [1^1^,2^1^:2^3^,3^1^:3^3^,4^1^-Quaterphenyl]-1^2^,1^4^,1^6^,2^2^,2^4^,2^6^,3^2^,3^4^,3^6^,4^2^,4^4^,4^6^-dodecol	Oligomer of phloroglucinols (a fucol-type phlorotannin)	Brown seaweeds	- Counteracting high free radicals production - Ability to activate the transformation of white adipose tissue to “brown” - Tackling neurodegeneration	[79] [80] [193]
Rosmarinic acid	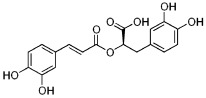 (2*R*)-3-(3,4-Dihydroxyphenyl)-2-{[(2*E*)-3-(3,4-dihydroxyphenyl)prop-2-enoyl]oxy}182propanoic acid	Hydroxycinnamic acids	Mainly *Lamiaceae* plants (especially from the *Nepetoideae* subfamily)	- Antioxidant and anti-inflammatory actions, - Ability to decrease the blood glucose, triglyceride, and plasma total cholesterol levels significantly	[147] [187] [215]
Hydroxytyrosol	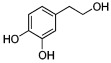 4-(2-Hydroxyethyl)benzene-1,2-diol	Phenylethanoid (phenolic compound)	Olive oil (in the form of oleuropein)	- Inhibiting oxidative stress and inflammation, - Improving MtS parameters in case of excessive body weight, insulin resistance, and hypertension	[216] [217]
Chlorogenic acid	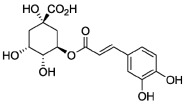 (1*S*,3*R*,4*R*,5*R*)-3-{[(2*E*)-3-(3,4-Dihydroxyphenyl)prop-2-enoyl]oxy}-1,4,5-trihydroxycyclohexane-1-carboxylic acid	Hydroxycinnamic acids (phenolic compound)	Coffee beans, peaches, eggplant, prunes	- Anti-inflammatory, antioxidant, anticarcinogenic activities - Hypoglycemic and hypolipidemic effects	[43] [218]
Caffecic acid	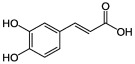 3-(3,4-Dihydroxyphenyl)-2-propenoic acid3,4-Dihydroxycinnamic acid	Hydroxycinnamic acids (phenolic compound)	Coffee beans, *Lamiaceae* plants, etc.	- Antioxidant, anti-inflammatory, anticancer and antidiabetic properties, - Ability to reverse insulin resistance, dyslipidemia, hyperglycemia, and oxidative stress in case of MtS	[43] [219] [220]
Ferulic acid	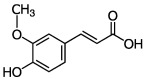 (2*E*)-3-(4-hydroxy-3-methoxyphenyl)prop-2-enoic acid	Hydroxycinnamic acids (phenolic compound)	Mainly *Apiaceae* plants (*Angelica sinensis*, genus *Ferula, etc.*)	- Lowering stored fat in human adipocytes, - Reversing insulin resistance, dyslipidemia, hyperglycemia, inflammation, and oxidative stress	[207] [220] [221]

## Data Availability

Not applicable.
